# Venous Thromboembolism in Critical Illness and Trauma: Pediatric Perspectives

**DOI:** 10.3389/fped.2017.00047

**Published:** 2017-03-13

**Authors:** Ranjit S. Chima, Sheila J. Hanson

**Affiliations:** ^1^Division of Critical Care Medicine, Cincinnati Children’s Hospital Medical Center, Department of Pediatrics, University of Cincinnati College of Medicine, Cincinnati, OH, USA; ^2^Medical College of Wisconsin, Children’s Hospital of Wisconsin, Milwaukee, WI, USA

**Keywords:** venous thromboembolism, deep vein thrombosis, pediatric critical illness, pediatric trauma, child, prophylaxis

## Abstract

Critically ill children and those sustaining severe traumatic injuries are at higher risk for developing venous thromboembolism (VTE) than other hospitalized children. Multiple factors including the need for central venous catheters, immobility, surgical procedures, malignancy, and dysregulated inflammatory state confer this increased risk. As well as being at higher risk of VTE, this population is frequently at an increased risk of bleeding, making the decision of prophylactic anticoagulation even more nuanced. The use of pharmacologic and mechanical prophylaxis remains variable in this high-risk cohort. VTE pharmacologic prophylaxis is an accepted practice in adult trauma and intensive care to prevent VTE development and associated morbidity, but it is not standardized in critically ill or injured children. Given the lack of pediatric specific guidelines, prevention strategies are variably extrapolated from the successful use of mechanical and pharmacologic prophylaxis in adults, despite the differences in developmental hemostasis and thrombosis risk between children and adults. Whether the burden of VTE can be reduced in the pediatric critically ill or injured population is not known given the lack of robust data. There are no trials in children showing efficacy of mechanical compression devices or prophylactic anticoagulation in reducing the rate of VTE. Risk stratification using clinical factors has been shown to identify those at highest risk for VTE and allows targeted prophylaxis. It remains unproven if such a strategy will mitigate the risk of VTE and its potential sequelae.

## Introduction

Venous thromboembolism (VTE) diagnosis in hospitalized children appears to have increased markedly over the past decade (1). Critically ill and/or severely injured children are at a disproportionately higher risk of VTE events due to the presence of multiple VTE risk factors (2, 3). Clinical diagnosis of VTE can be especially challenging in a critically ill and severely injured child as extremity swelling and erythema may be non-specific signs and self-reporting of pain is limited by sedation, immobility, and physical state. Hence, a high degree of suspicion is needed on the part of a clinician to perform imaging and diagnose VTE.

In critically ill and injured adults, mechanical and pharmacologic prophylaxis is an accepted practice to mitigate VTE (4); however, this is not the case in children. In addition, VTE risk prediction and stratification remains a challenge and robust risk scoring systems remain elusive in children. Even if one were to develop the perfect risk screen, there are no data showing a benefit from prophylaxis in critically ill children. Despite the paucity of evidence surrounding screening and prophylaxis, health-care providers are motivated to develop strategies to reduce the incidence of VTE in children. While mechanical prophylaxis is relatively risk free, pharmacologic prophylaxis may increase the bleeding risk in this group of patients. This review will summarize the epidemiology/incidence and current controversies in regards to VTE in critically ill and severely injured children.

## Incidence and Risk Factors for VTE in Critically Ill or Injured Children

An increase in the diagnosis for VTE has been reported for hospitalized children from 2001 to 2008 (1); however, it is not known if this increase is equivalent among the subpopulations of critically ill or injured children. The interpretation of reported rates of VTE is confounded by the lack of standard VTE screening or for diagnosis of VTE. The reported incidence of VTE in critically ill and/or severely injured children is summarized in Table 1. There is a wide variation in incidence based on the study design and the specific population included. For instance, the incidence of VTE in pediatric trauma ranges from 0.2 to 0.5% in large, retrospective studies of hospitalized children using databases (5–10), while higher rates of 0.9–1.1% were found in smaller studies using data from the patient records (11, 12). For injured children admitted to the intensive care unit (ICU), the incidence rate (0.3–6.2%) is higher especially in prospective studies (3, 13–15).

**Table 1:** **Incidence of VTE in critically ill or injured children**.^a^

Reference	Design/data source	Population	VTE (*N*/total)	VTE incidence (%)
Allen et al. (11)	Retrospective, single center	Trauma	22/1,934	1.1
Connelly et al. (5)	Retrospective, NTDB data	Trauma	1,141/536,423	0.2
Yen et al. (8)	Retrospective, single center and NTDB data	Trauma	Single center: 49/17,366	0.3–0.4
			NTDB 2011–2012: 1,168/281,248	
Carpenter et al. (22)	Retrospective, single center	ICU (bacteremia)	21/229	9.2
Arlikar et al. (16)	Retrospective, single center, case-control, ICD-9 codes	ICU	57/19,000 (est)	0.3
Harris and Lam (10)	Retrospective, KID data	Trauma (TBI)	267/58,529	0.5
Al Tassan et al. (19)	Retrospective, single center	ICU (CVC)	21/248 CVC	8.5
Van Arendonk et al. (6)	Retrospective, NTDB data	Trauma	1,655/402,329	0.4
Faustino et al. (18)	Prospective, multicenter	ICU (CVC)	16/101	15.8
Askegard-Giesmann et al. (7)	Retrospective, multicenter, PHIS data	Trauma	671/260,078	0.3
O’Brien et al. (12)	Retrospective, multicenter, local trauma registries	Trauma	15/1,706	0.9
Hanson et al. (14)	Prospective, single center	Trauma, ICU	3/169	1.7
Greenwald et al. (30)	Retrospective, single center	Trauma	3/1,782	0.2
Hanson et al. (21)	Nested case–control, single center	ICU (cardiac disease)	41/1,070	3.8
Higgerson et al. (17)	Prospective, multicenter	ICU	62/6,653	0.9
O’Brien and Candrilli (13)	Retrospective, multicenter NTDB data	Trauma, ICU	1,087/135,032	0.8
Hanson et al. (15)	Nested case–control, single center	Trauma, ICU	9/144	6.2
Hanslik et al. (20)	Prospective, single center	ICU (CVC and cardiac disease)	25/90	27.8
Candrilli et al. (9)	Retrospective, multicenter, HCUP-KID data	Trauma	648/240,387	0.3
Cyr et al. (3)	Retrospective, single center, ICD-9 data	Trauma, ICU	11/3,291	0.3

*^a^Age <21 years, includes only studies published since 2006 with defined incidence of VTE in pediatric ICU or in trauma populations*.

*NTDB, National Trauma Data Bank; TBI, traumatic brain injury; CVC, central venous catheter; KID, Kids Inpatient Database; PHIS, pediatric health information system; VTE, venous thromboembolism; ICU, intensive care unit*.

For the general pediatric ICU population, the incidence of VTE ranges 0.3–0.9% (16–22), with the higher incidence being reported from prospective studies. Specific subpopulations of critically ill children report higher incidence of VTE including those with central venous catheters (CVCs) (17–19), cardiac disease (20, 21), and bacteremia (22).

The risk factors for VTE in critically ill and/or severely injured children are summarized in Table 2. Critically ill and/or severely injured children are at a disproportionately higher risk of VTE events than any other cohort of children due to the presence of multiple risk factors: including endothelial injury from trauma, CVC placement, and/or operative procedures; alterations in blood flow from immobility and poor perfusion requiring inotropic support; and hypercoagulability from sepsis, trauma, blood transfusion, or other dysregulated inflammatory states (see Table 2). The exact contribution of each of these factors is unclear; however, the presence of a CVC is probably the most important risk factor for VTE in this cohort of children. Most, but not all, studies found increasing age to be a risk factor for VTE in injured and critically ill children (see Table 2). In studies that performed a separate analysis for infants, <1 year of age was also associated with increased risk. For trauma patients, the risk of VTE appears to increase with higher injury severity scores (ISS) (see Table 2).

**Table 2:** **Risk factors for VTE in critically ill or injured children**.

Reference	Age (years)	CVC	Surgery	Illness/injury severity	Other risk factors
Allen et al. (11)	>13, OR 9.2	OR 4.4	Orthopedic, OR 6.8	N/A	MVI, OR 15.4

Harris and Lam (10)	>15, OR 3.7	OR 3.0	Orthopedic, OR 2.44	N/A	Ventilator, OR 1.9
			Cranial, OR 1.78		Tracheostomy, OR 2.3
					NAT, OR 2.8

Yen et al. (8)	13–15, OR 3.81	N/A	OR 8.0	ISS 9–15, OR 4.1	GCS < 9, OR 2.8
	>16, OR 5.22			ISS 16–24, OR 10.8	Transfusion, OR 2.8
				ISS > 25, OR 15.7	

Carpenter et al. (22)	NS	NS	N/A	N/A	CRP > 20, OR 4.2
					Hg nadir < 9, OR 5.2

Connelly et al. (5)	13–15, OR 1.3	OR 1.9	OR 4.5	N/A	ICU, OR 5.5
	16–17, OR 1.7				Ventilator, OR 2.6
					GCS < 9, OR 1.4
					Pelvic/LE fx, OR 1.4

Arlikar et al. (16)	NS	OR 26	NS	N/A	Infection, OR 3.4

Van Arendonk et al. (6)	13–15, OR 2.0	OR 1.3	OR 3.8	ISS 9–15, OR 3.9	Ventilator, OR 2.5
	>16, OR 3.8			ISS 16–24, OR 5.9	Transfusion, OR 1.5
				ISS > 25, OR 7.2	GCS < 9, OR 1.3

Faustino et al. (18)	>13, OR 14.1	All	Postop-NS	PIM2-NS	

Askegard-Giesmann et al. (7)	N/A	OR 8.0	N/A	N/A	ICU OR, 3.7
					Pelvic fx OR, 1.6

Hanson et al. (21)	NS	OR 1.1	N/A	PRISM3-NS	Single ventricle, OR 11.2

Higgerson et al. (17)	N/A	OR 9.3	N/A	N/A	

O’Brien and Candrilli (13)	<1, OR 1.75	OR 1.8	Cranial, OR 1.8	N/A	TBI, OR 1.33
	14–17, OR 2.34		Open LE, OR 1.1		LE fx, OR 1.8
			Vascular, OR 2.8		Pelvic fx, OR 1.2

Hanson et al. (15)	NS	OR 19	N/A	NS	PN, OR 20.8
					NMB, OR 10.0
					Inotropes, OR 10

Candrilli et al. (9)	NA	NA	NA	ISS 9–15, OR 2.1	
				ISS 16–25, OR 2.5	
				ISS > 25, OR 3.5	

Cyr et al. (3)	15–18, OR 19.5	OR 64	Chest, OR 6.9	ISS > 8, OR 5.3	SCI, OR 37.4

*OR, adjusted odds ratio; NS, not significant; NA, not analyzed; MVI, motor vehicle injury; ISS, injury severity score; NAT, non-accidental trauma; CRP, C-reactive protein; Hg, hemoglobin; CVC, central venous catheter; LE fx, lower extremity fracture; GCS, Glasgow Coma Scale; PIM2, paediatric index of mortality 2; PRISM3, pediatric risk of mortality score; TBI, traumatic brain injury; NMB, neuromuscular blockade; PN, parenteral nutrition; SCI, spinal cord injury; ICU, intensive care unit*.

Overall interpretation and generalizability of data in regards to incidence and risk factors is limited, given the significant differences in the population included and study design. Several, recent, large studies in children have used diagnostic codes for the identification of VTE (5, 6). Using diagnostic codes for identification of pediatric VTE has a low specificity and sensitivity (23). Hence, misidentification of children with and without VTE could result in differences in incidence rates and risk factors. Likewise, studies with smaller numbers of patients may fail to identify significant risk factors. Despite these limitations in study populations and methodologies, the incidence of VTE appears to increase in patients with multiple risk factors, with the presence of a CVC being the most important risk factor in critically ill and/or injured patients. Certain subpopulations of critically ill children have a greater risk for VTE, with an incidence of VTE >1%.

## Prevention of VTE in Critically Ill or Injured Children

Efforts for prevention of VTE in critically ill or injured patients hinge on early mobilization and the use of mechanical and/or pharmacologic prophylaxis. Mechanical prophylaxis includes the use of sequential compression devices (SCD) or graduated compression stockings, both of which are limited by size and cannot be used in smaller children and on injured extremities. There are no pediatric studies showing efficacy of mechanical prophylaxis in preventing VTE.

There is little evidence to guide the use of pharmacologic thromboprophylaxis in critically ill and injured children. Published pediatric guidelines are based on weak evidence and recommend against the use of pharmacologic prophylaxis except in children with cyanotic congenital heart disease, dilated cardiomyopathy, cavopulmonary anastomosis, end-stage renal disease, and primary pulmonary hypertension (24). A recently published consensus of experts in regards to pediatric trauma recommended against prophylaxis in children <12 years of age and gave a strong recommendation for pharmacologic prophylaxis in patients with a history of VTE, while a weak recommendation for patients with CVCs (25). Given the lack of data, it is not surprising that there is a wide variation in thromboprophylactic practices in critically ill children as shown in the PROTRACT study (26). This global point-prevalence study clearly demonstrated that the use of both mechanical and pharmacologic prophylaxes was center dependent with a wide variation in the use of prophylaxis. Data were collected on the type of pharmacologic thromboprophylaxis used in the ICU including aspirin, low-molecular-weight heparin, IV unfractionated heparin (UFH), subcutaneous UFH, warfarin, and clopidogrel. Aspirin was the most commonly used agent (143 of 308 patients, 46.4%), primarily because of patients with congenital heart disease. LMWH, almost exclusively enoxaparin, was the next most commonly used agent (113 of 308 patients, 36.7%). Warfarin was rarely used in the ICU setting (26).

Critically ill and/or injured children represent a high-risk cohort for VTE, especially in the setting of CVCs, and may merit from thromboprophylaxis. This is especially true as patients approach adulthood wherein heparin-based prophylaxis regimens have been shown to be effective in preventing VTE in critically ill adults (4). Whether such strategies are of benefit in critically ill and/or injured children remain unproven. However, a standardized systematic approach to VTE prevention may result in a reduction in VTE. This was demonstrated in a single-center study in the setting of pediatric trauma where a reduction in incidence of VTE was noted after implementation of standardized thromboprophylaxis guidelines (14). Notably in this study, the reduced incidence of VTE was not associated with an increase in pharmacologic prophylaxis. The authors speculate that the decrease in VTE was a result of standardized, focused pharmacologic prophylaxis to those patients at high risk for VTE.

Pharmacologic prophylaxis should be instituted thoughtfully especially in patients at high risk for bleeding. There are minimal data on bleeding in the setting of pharmacologic prophylaxis for VTE in the critical care or trauma setting in pediatrics. In a multicenter review of trauma registry data to assess pharmacologic prophylaxis, the rate of major bleeding was 0.3% (12). However, single-center data demonstrated a higher rate of 4% in hospitalized pediatric patients receiving pharmacologic prophylaxis (27). A recent prospective observational study of hospitalized children receiving prophylactic anticoagulation showed a similar incidence of major bleeding especially in patients following orthopedic surgery (28). Taken together, the data demonstrate a low but definite risk of bleeding children receiving pharmacologic prophylaxis. Hence, it is imperative that any preventive strategy utilizing pharmacologic prophylaxis account for the bleeding risk, especially in a high bleeding-risk cohort, such as children who are critically ill or severely injured.

In summary, VTE prevention in critically ill and/or injured children needs a standardized approach with VTE risk stratification. Interventions should include early mobilization and removal of CVCs alongside mechanical and pharmacologic prophylaxes, especially in children >12 years of age.

## Predicting VTE Risk in Children after Trauma

Recently, two scoring systems to predict the risk of VTE in children hospitalized after trauma have been developed (5, 8). Both studies used the National Trauma Data Bank to derive and validate the VTE risk score over similar time periods. The model from Connelly et al. had good performance with an area under the curve of 93–94% (5). This model incorporated 10 VTE risk factors: age (increased risk for <1 year and adolescence), sex, Glasgow Coma Scale (GCS), CVC, intubation, blood transfusion, ICU admission, major surgery, pelvic fracture, and lower extremity fracture. Varying points for each risk factor are summed for a total score. Categorical risk was assigned based on this score: low risk (VTE incidence <1%), medium risk (VTE incidence 1–5%), and high risk (VTE incidence >5%). The authors suggest a potential management strategy to implement screening ultrasounds and SCD for the medium-risk group, with the addition of pharmacologic prophylaxis for the high-risk group. By contrast, Yen et al. used a combination of local trauma registry data and the national trauma data bank for development and validation of a VTE risk score model with good performance as shown by the area under curve of 91% (8). The preferred model incorporates six risk factors, for which varying points are accumulated: older age, GCS, ISS, blood transfusion, intubation, and major surgery. CVC was not analyzed as a risk factor for the model. A score >17 is associated with VTE risk >2%, referenced as a threshold for prophylaxis.

These studies provide the framework to convert epidemiologic risk factors into tools clinicians can use to predict the overall VTE risk for their injured patient. Both studies recognize the limitations of the national trauma database: surveillance bias, no temporal association of risks (intubation, surgery, and transfusion) with the development of VTE, and the confounding effect of variable use of thromboprophylaxis. The rare occurrence of VTE in the overall hospitalized pediatric trauma population makes large database studies necessary to provide adequate power of associated risks, with the risks studied limited to those captured in the database. As injured children in the ICU have a higher VTE rate compared to the overall hospitalized children after trauma, this high-risk population may be appropriate to prospectively validate and refine an optimal VTE prediction tool.

## Future Directions

The ever-increasing medical complexity of critically ill and injured children implies that the risk of VTE will continue to be present especially in the setting of CVCs. Hence, standardized risk prediction and stratification will be the key to implementing any thromboprophylactic strategy. Validation of risk prediction tools will be challenging given the low overall incidence for VTE in children. Currently, most risk assessment algorithms use clinical variables, and whether the addition of biomarkers bolsters their performance remains unclear. A recent prospective study in critically ill children with CVCs showed an association between factor VIII activity and catheter-related thrombosis (29).

Even with the ideal risk prediction tool, the appropriate interventions to prevent VTE are unknown. Hence, there is a pressing need to evaluate the efficacy of interventions in preventing VTE in children, including pharmacologic or mechanical prophylaxis, early ICU rehabilitation, and increased mobility. Given the low incidence of VTE in children, focusing on the subpopulations of critically ill or injured children at highest risk for VTE, including those with CVCs, will optimize the results of any clinical trial.

## Author Contributions

RC and SH conceptualized and wrote the manuscript.

## Conflict of Interest Statement

The authors declare that the research was conducted in the absence of any commercial or financial relationships that could be construed as a potential conflict of interest.
